# The Effect of H_2_S Pressure on the Formation of Multiple Corrosion Products on 316L Stainless Steel Surface

**DOI:** 10.1155/2020/3989563

**Published:** 2020-07-26

**Authors:** M. Shah, M. T. M. Ayob, R. Rosdan, N. Yaakob, Z. Embong, N. K. Othman

**Affiliations:** ^1^DNVGL Malaysia Sdn. Bhd., Level 18, Menara Prestige, No. 1, Jalan Pinang, Kuala Lumpur 50450, Malaysia; ^2^Department of Applied Physics, Faculty of Science and Technology, The National University of Malaysia (UKM), Bangi 43600, Selangor, Malaysia; ^3^Center of Industrial Process Reliability and Sustainability (INPRES), Faculty of Chemical Engineering, Universiti Teknologi MARA (UiTM), Shah Alam 40450, Selangor, Malaysia; ^4^Faculty of Applied Science and Technology, Tun Hussein Onn University of Malaysia (UTHM), KM 1. Jalan Panchor, Muar 84600, Johor, Malaysia

## Abstract

H_2_S gas when exposed to metal can be responsible for both general and localized corrosion, which depend on several parameters such as H_2_S concentration and the corrosion product layer formed. Therefore, the formation of passive film on 316L steel when exposed to H_2_S environment was investigated using several analysis methods such as FESEM and STEM/EDS analyses, which identified a sulfur species underneath the porous structure of the passive film. X-ray photoelectron spectroscopy analysis demonstrated that the first layer of CrO_3_ and Cr_2_O_3_ was dissolved, accelerated by the presence of H_2_S-Cl^−^. An FeS_2_ layer was formed by incorporation of Fe and sulfide; then, passivation by Mo took place by forming a MoO_2_ layer. NiO, Ni(OH)_2_, and NiS barriers are formed as final protection for 316L steel. Therefore, Ni and Mo play an important role as a dual barrier to maintain the stability of 316L steel in high pH_2_S environments. For safety concern, this paper is aimed to point out a few challenges dealing with high partial pressure of H_2_S and limitation of 316L steel under highly sour condition for the oil and gas production system.

## 1. Introduction

Southeast Asia is a rich source of natural gas and petroleum, especially Brunei, Vietnam, Malaysia, and Indonesia. From GlobalData statistic, Malaysia and Indonesia will contribute around 80% and 70% of the Southeast Asia's total crude oil and natural gas production from eight planned projects in 2025, respectively. In brief, once the well is successfully drilled and installation is completed, the product must be transported to a facility where it can be treated, stored, processed, and refined through the pipeline system. Therefore, the environmental conditions of the production and pipeline systems are required for the prediction of materials life cycle and its maintenance requirements. Thus, any corrosion that occurs inside oil and gas pipeline systems is a serious factor that could lead to system failure. Corrosion not only causes economic losses but also greatly affects the safety and protection of the oil and gas resource. Hence, in upstream oil and gas applications, the most desirable alloy should be made of a strong material with good localized corrosion resistance, low cost, and suitable mechanical characteristics.

Austenitic stainless steel type 316L (UNS S31603) has excellent corrosion resistance to the electrochemical properties of the passive film that forms on its surface. The passive film mainly consists of iron (Fe), chromium (Cr), and nickel (Ni) oxides. Cr promotes the formation of protective surface oxide, and Ni enhances the stability of the oxide film. Thus, stainless steel alloys with a higher composition of Cr and Ni prevent the iron from rusting and provide heat resistant properties [1]. Besides, 316L steel is a low carbon alloy that contains molybdenum (Mo), which also makes it more corrosion resistant, especially in highly sour conditions [1].

The physicochemistry of passive films directly influences the film properties. Therefore, many studies have investigated the effect of hydrogen sulfide corrosion at different hydrogen sulfide partial pressures (pH_2_S) [2], pH [3], and temperature [4] on the material surface. However, this area remains underexplored due to the cross effects between the parameters of the study [3]. Ding et al. [1] investigated the electrochemical behavior of 316L steel in Cl^−^ solutions under different pH_2_S. Additionally, Gao et al. [4] found an increased presence of iron oxide (Fe_3_O_4_) and iron sulfide in the corrosion product layer on substrate after performing H_2_S corrosion tests involving increased exposure times at 120°C. When both H_2_S and Cl^−^ were present, the passive film would become more sensitive to temperature, therefore significantly affecting the corrosion rate, the corrosion mechanism, and the properties of the material surface. Although various corrosion reactions could occur, the corrosion rate generally increases in the presence of H_2_S until sulfide saturation was achieved [2, 5].

Therefore, the authors aim to determine the effect of different pH_2_S exposure on the formation of multiple corrosion product layer of the 316L steel surface. The surface analysis of 316L steel was performed using a multianalytical technique consisting of X-ray photoelectron spectroscopy (XPS), field emission scanning electron microscopy (FESEM), and energy dispersive X-ray spectrometry (EDS). The microstructure characterization and crystalline phase characterization of 316L steels were performed using transmission electron microscopy (TEM). The metal ions that had dissolved in the test solution after 7 days were characterized via inductively coupled plasma-mass spectrometry (ICP-MS). Finally, the reaction mechanism occurring on the surface of 316L steel was explained in terms of the dissolution of CrO_3_ and Cr_2_O_3_ on the steel protective film at 3 bar pH_2_S after 7 days of testing.

## 2. Experiment

### 2.1. Materials and Method

For the experiment, a commercial austenitic stainless steel type 316L with a chemical composition listed in Table 1 was used. Then, optical emission vacuum spectrometer analysis was performed on the stainless steel using the point-to-plane excitation technique, following ASTM E 1086 Standards [6]. The 316 L steel sample was machined into a dimension of 20 × 20 × 3 mm^3^, abraded with a series of silicon carbide papers (up to 1200 grit), polished with a diamond spray (up to 1.0 *μ*m), rinsed with distilled water, degreased with acetone, dried at room temperature, and then stored in a desiccator. Each procedure for preparing the 316L steels was performed following ASTM G1-03 Standards [7]. All experiments were carried out in NACE TM0177 Solution A [8]. The solutions were added to an autoclave and deoxygenated by purging with nitrogen gas for two hours before the beginning of each experiment. All experiments were conducted at 60°C and pH 3.0 ± 0.5 to simulate the crude oil conditions in Southeast Asia especially around Malaysian region (30°C–90°C and pH 2–6) [9].

### 2.2. Experimental Procedure and Weight-Loss Measurement

**Figure 1:**
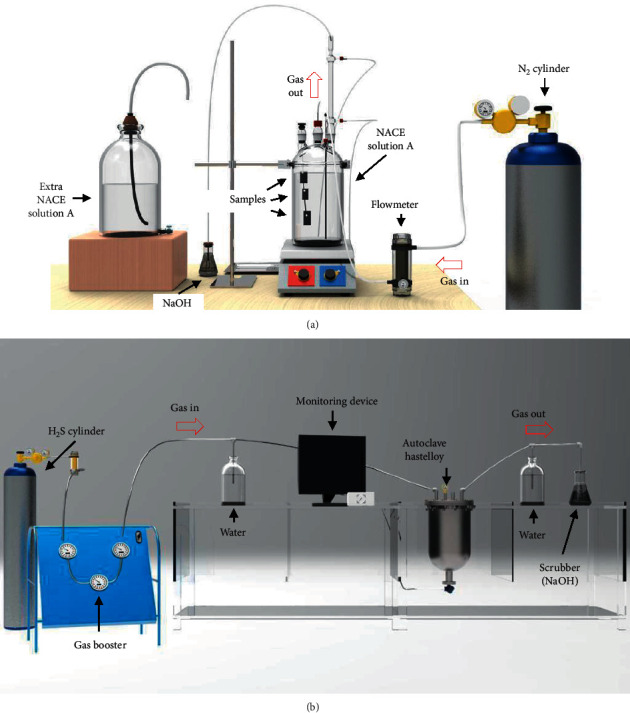
Schematic diagram of the corrosion test apparatus for (a) 0 bar and (b) 3 bar of the partial pressure H_2_S test.

Experiments were carried out in a 5 L autoclave using experimental conditions which are summarized in Table 2. A schematic diagram of the corrosion test apparatus was set up for the 0 bar and 3 bar of pH_2_S and is shown in Figures 1(a) and 1(b), respectively, in Sour lab, at DNVGL Singapore laboratory. The samples were weighed using an analytical balance for the initial weight (*W*_i_), before being immersed vertically in test solution. Then, the 316L steel sample with a holder was attached to the top lid and sealed in the autoclave before immersing it vertically in the test solution. Besides, 3.5 L of the NACE A solution and 1.5 L vapor were used to allow the water vapor to expand in high pressure and temperature conditions. After that, a deoxygenation process was conducted by purging the autoclave with nitrogen gas for two hours before each test. The samples were heated at 60°C and pressurized with premixed gases (3 bar of H_2_S and 27 bar of N_2_ gas) equaling a total pressure of 30 bar until a stable pressure was reached. The pH and temperature of the solution were measured and recorded at the start and end of each experiment as shown in Table 2. After 7 days of immersion, the samples were taken out from the solution, cleaned, and preserved with nitrogen according to ASTM G1-03 Standards [7]. The final samples were weighed again to obtain the final weight (*W*_f_) based on which weight loss was determined. After that, the corrosion rate was calculated using the following equation:

(1) $$\begin{array}{c} \text{corrosion rate}=\;\frac{kW}{pAT\;}, \end{array}$$

where *K* = constant, *W* = weight loss in mg, *ρ* = density of the metal in g/cm^3^, *A* = area in cm^2^, and *T* = time of exposure in hours. Then, the procedure was repeated with H_2_S-free conditions for the control sample in this experiment.

### 2.3. Characterization of Corrosion Products

Morphological images characterization of all 316L steels were performed using a Zeiss SUPRA 55VP field emission scanning electron microscope (FESEM) and a high-efficiency in-lens detector for clear topographic imaging in high-vacuum mode, in conjunction with energy dispersive X-ray spectroscopy (EDS) to characterize the elemental composition of the samples. The surface analysis was performed using X-ray photoelectron spectroscopy (XPS) (X-ray microprobe PHI Quantera II, Ulvac-PHI, Inc.) with a monochromatic Al-K*α* (hv = 1486.6 eV) X-ray source. Before deconvolution, the charging effects were corrected using a Kratos charge neutralizer system in order to minimize the carbon charging effect. In this case, all spectrum is corrected and referred to adventitious carbon binding energy at 284.8 eV. A TEM thin foil was prepared using the focused ion beam (FIB) technique (Helios Nanolab 600i, FEI). Also, detailed subsurface microstructure characterization was performed on a transmission electron microscope (TEM) (Tecnai G2 F20, FEI) with an EDS detector operated at 200 kV. Finally, the elemental analyses of the corrosion product dissolution in the test solutions were carried out on an inductively coupled plasma-mass spectrometer (ICP-MS) PerkinElmer Sciex Elan 9000.

## 3. Results and Discussion

### 3.1. The Effect of H_2_S Pressure on the Corrosion Rate

The average corrosion rate of 316L steels after immersion in test solution at 0 bar and 3 bar pH_2_S were calculated using weight loss measurement. The 316L steel at 3 bar pH_2_S had higher corrosion rate compared to that in 0 bar pH_2_S of 316L steel. The corrosion rate increased by 90.1% from 0.07 mm/yr at 0 bar pH_2_S to 0.74 mm/yr at 3 bar pH_2_S due to aggressiveness of sulfide ions in test solution [1]. The data show that overall mass loss is symmetrical with H_2_S pressure, and sulfide ions play a significant role in determining the kind of corrosion scales and reducing the surface protection of 316L steel [1].

### 3.2. FESEM-EDS

**Figure 2:**
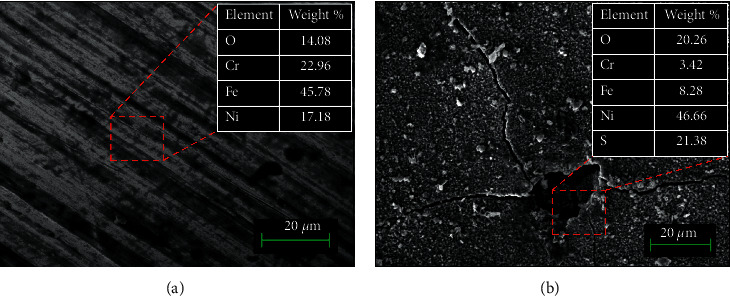
Surface morphology image and elemental concentration analysis using SEM-EDS of 316L austenitic stainless steel at (a) 0 bar and (b) 3 bar pH_2_S at 60°C using a magnification of 10 *µ*m.

The surface morphology of the austenitic 316L steels immersed in test solution without H_2_S at 60°C for 7 days is shown in Figure 2(a). Under an environment free of H_2_S, the 316L steel surface showed scratch marks because of polishing, but no grains or pits are shown. Besides, the elemental composition of 316L steel free of H_2_S was done using EDS, as shown in Figure 2(a). The surface of the 316L steel in the H_2_S-free conditions showed that the passive film was formed in the presence of Cr. However, when the 3 bar pH_2_S was applied, the surface of the 316L steel shows a cracked surface, which indicates local breakdown of the passive layer, as shown in Figure 2(b). The sulfide compounds were detected on the 316L steel surface as a result of applying pH_2_S. Due to local breakdown of the protective layer, microcracking was observed, as shown in Figure 2(b). Therefore, corrosion products are formed after 3 bar pH_2_S exposure, demonstrating that the protectiveness of the passive film had very much degraded. The EDS analysis in Figure 2(b) shows the presence of a sulfur element, suggesting the presence of metal sulfides layer on the 316L steel surface [10]. However, Cr signals were depleted in the protective layer after exposure with H_2_S due to the dissolution of Cr in the test solution, as reported by Beverskog and Puigdomenech (1997) [11].

### 3.3. X-Ray Photoelectron Spectroscopy (XPS)

**Figure 3:**
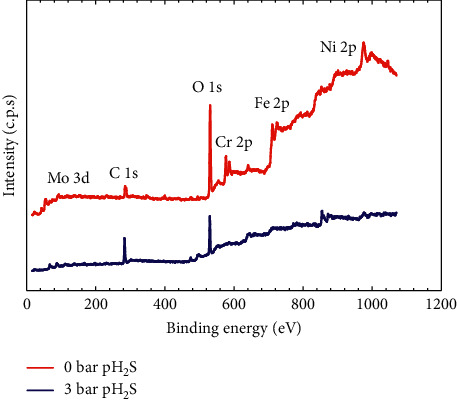
XPS wide spectra of the passive films formed on 316L austenitic stainless under the condition 0 bar pH_2_S in NACE A solution and 3 bars pH_2_S in NACE A solution.

**Figure 4:**
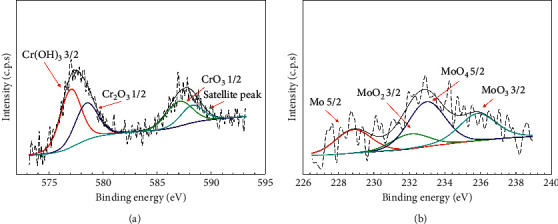
XPS spectra deconvolution of passive films formed on 316L austenitic stainless steel for a narrow scan of (a) Cr 2p and (b) Mo 3d at 60°C at 0 bar pH_2_S in NACE A solution.

The chemical composition of the 316L steel passive films that formed under different pH_2_S conditions after 7 days of immersion in test solution was investigated using XPS measurements, as shown in Figure 3. The major peaks present in the spectra for both 316L steels corresponded to C, O, Fe, Cr, Ni, Mo, and S. Generally, austenitic stainless steel is self-passivated, as chromium oxide (CrO_3_) instantly forms when it is exposed to air, but the nature of the passive layer changes when exposed to an aqueous solution [12]. Apart from that, the element contribution analysis of Cr and Mo was performed on the sample without exposure to H_2_S as shown in Figure 4. The Cr element is known as a key element in passive film formation. However, Figure 4(a) shows only three constituent peaks in the Cr 2p signal, representing Cr(OH)_3_ (Cr^3+^ 2p_3/2_; 577.5 eV), Cr_2_O_3_ (Cr^6+^ 2p_1/2_; 578.8 eV), and CrO_3_ (Cr^3+^ 2p_1/2_; 587.1 eV) formed during pre-exposure (during immersion) and Cr_2_O_3_ (577.1 eV) formed via the anion-cation reaction during postexposure (after immersion). The absence of Cr(OH)_3_ in this analysis implies that Cr was easily oxidized at lower pH (∼3.5) in H_2_S-free conditions. Besides, Figure 4(b) shows the narrow scan spectra of Mo 3d obtained on the sample in 0 bar pH_2_S conditions. The spectra of the doublet Mo (3d_3/2_ and 3d_5/2_) can be split into two components, corresponding to the oxides formed of Mo/MoO_2_ (Mo^4+^ 3d_5/2_; 228.8 eV and Mo^4+^ 3d_3/2_; 232.6 eV) and MoO_4_/MoO_3_ (Mo^6+^ 3d_5/2_; 232.9 eV and Mo^6+^ 3d_3/2_; 235.8 eV), respectively. It could also be observed that MoO_3_ was the primary Mo species.

**Figure 5:**
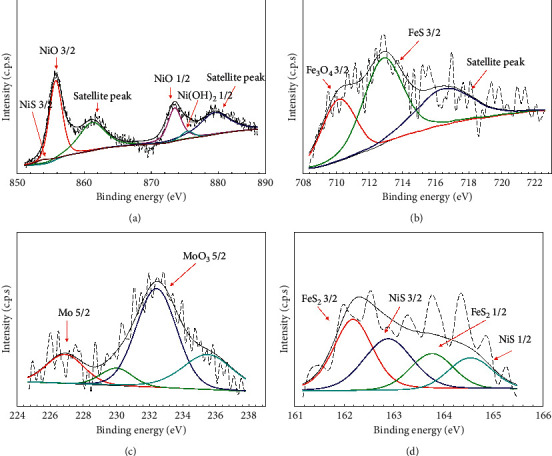
XPS spectra deconvolution of passive films formed on 316L austenitic stainless steel for a narrow scan of (a) Ni 2p, (b) Fe 2p, (c) Mo 3d, and (d) S 2p at 60°C with 3 bar pH_2_S in NACE A solution.

Meanwhile, the elemental contribution analysis of the specimen after immersion in the test solution in H_2_S-containing conditions yielded Ni, Fe, Mo, and S, as shown in Figure 5. The Ni 2p narrow spectra consisted of three compounds, namely, Ni(OH)_2_ (857.4 eV), NiO (855.7 eV), and NiS (853.9 eV), as shown in Figure 5(a). The dominant peaks of Ni 2p_3/2_ in the spectrum corresponded to NiO, indicating that NiO could stably exist in the passive film. However, Figure 5(b) shows the narrow scan spectra of Fe 2p_3/2_ obtained on the 316L steel sample, which was immersed in the H_2_S solution at 3 bar pH_2_S for 7 days. Two deconvolution signals were observed, attributed to Fe_3_O_4_ (710.1 eV) and FeS (712.8 eV). From the reactivity series, Fe was more active than Ni and could participate in the passive film formation in the form of Fe_3_O_4_, Fe_2_O_3_, Fe(OH)_2_, FeOOH, and Fe(OH)_3_ [13]. However, iron sulfide (FeS) also appeared in the film due to the high affinity of sulfides with iron in the H_2_S solution [14]. Moreover, the possibility of Fe species dissolved preferentially in the H_2_S-containing solution and affected the migration of S^2−^ into the passive film, preventing the formation of Fe_2_O_3_ on the passive film [13]. Apart from that, the Mo 3d_3/2_ spectra consisted of MoO_3_ (3d_3/2_; 235.5 eV and 3d_5/2_; 232.4 eV), MoO_2_ (3d_3/2_; 230.2 eV), and bulk Mo from metal (226.9 eV), as shown in Figure 5(c). This result is similar to Wang et al. (2017), which reported a Mo 3d photoelectron peak binding energy position at 232.6 eV and 235.8 eV, corresponding to molybdenum oxides (MoO_3_) [15]. Although the H_2_S molecule has a chemical structure similar to H_2_O, the polarizability of the sulfides such as S^2−^ or HS^−^ is higher than that of halides (Cl^−^) and OH^−^ when adsorbed onto the passive film and could appear in that film [16]. Since chromium sulfides were not detected, S^2-^ could only unite with Fe^2+^ and Ni^2+^. Therefore, from the deconvolution of S 2p shown in Figure 5(d), four peaks were fitted by the narrow spectra at the binding energy of FeS_2_ (2p_3/2_; 162.2 eV), NiS (2p_3/2_; 162.9 eV), FeS_2_ (2p_1/2_; 163.7 eV), and NiS (2p_1/2_; 164.5 eV) corresponding to disulfide (S_2_^2−^), S^2−^, and S, respectively [16]. Based on the reactivity series of metals, nickel is less reactive than Cr and Fe, but nickel oxide could be porous and could easily entrap S^2-^ within the porous oxide. However, traces of NiO and/or Ni(OH)_2_ were also found in the passive film, similar to that reported by Luo [17].

**Figure 6:**
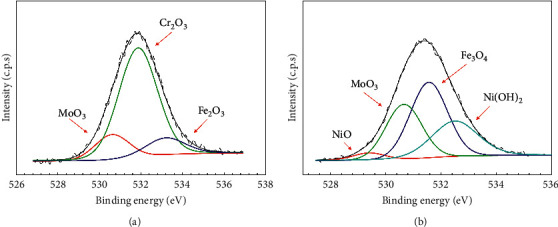
XPS narrow scan deconvolution of passive films formed on 316L austenitic stainless steel for (a) O 1s at 60°C 0 bar pH_2_S and (b) O 1s 60°C with 3 bar pH_2_S in NACE A solution.

The O 1s narrow scan spectra for austenitic 316L steel for both 0 bar and 3 bar H_2_S-containing conditions are shown in Figure 6. From the deconvolution of O 1s, several oxide species were found on the samples with O^2-^ constituents in H_2_S free conditions attributed to Cr_2_O_3_ (531.9 eV) and MoO_3_ (530.6 eV), as shown in Figure 6(a). Meanwhile, Figure 6(b) shows the O 1s spectra of the sample in the 3 bar pH_2_S-containing conditions being dominated by Ni(OH)_2_ (532.5 eV)_,_ Fe_3_O_4_ (531.6 eV), MoO_3_ (530.7 eV), and NiO (529.4 eV), formed via the lattice nonconserving reaction through diffusion vacancy or interstitial vacancy filling based on the cation-anion reaction [18]. Besides, the Cr content in the passive film was very low, thus having no corresponding XPS spectra and revealing a decreased stability of this species at high H_2_S-Cl^−^ concentration in the test solution.

Based on the XPS deconvolution in the atomic concentration analysis of Table 3, 316L with H_2_S-free conditions showed signals attributed to Mo, Cr, and O. Meanwhile, Cr and Mo compounds were found associated with O to form Cr_2_O_3_, CrO_3_, MoO_3,_ and MoO_2_. Besides, the sample in H_2_S conditions produced more signals corresponding to Mo, Ni, Fe, S, O, and C. The atomic concentration of Cr was reduced after exposure to 3 bar pH_2_S that could be due to the adsorption and dissociation of sulfide ions (HS^−^ and S^2-^) on 316L steel surface, and then react with chromium oxides (Cr_2_O_3_ and CrO_3_) [19]. The Mo compound showed MoO_3_ signals with a photoelectron signal of 232.41 eV and metallic Mo with a photoelectron signal of 226.91 eV. The presence of sulfide contributed to the signals of FeS_2_ and NiS on the sample surface. Aside from that, the Ni signals were attributed to NiO, Ni(OH)_2_, and NiS. The product of the final chemisorption process was derived from the Fe elements, namely, Fe_3_O_4_ and FeS_2_. The summary of corrosion product layer formed at each condition is shown in Table 4. The corrosion product formed at 0 bar H_2_S consists of Cr_2_O_3_, CrO_3_, MoO_3,_ and MoO_2_. Whilst the corrosion product at 3 bar H_2_S consists of the multiple corrosion product layer including Mo, MoO_3_, NiS, Ni(OH)_2_, NiO, FeS, and Fe_3_O_4_.

### 3.4. Inductively Coupled Plasma-Mass Spectrometry (ICP-MS)

**Figure 7:**
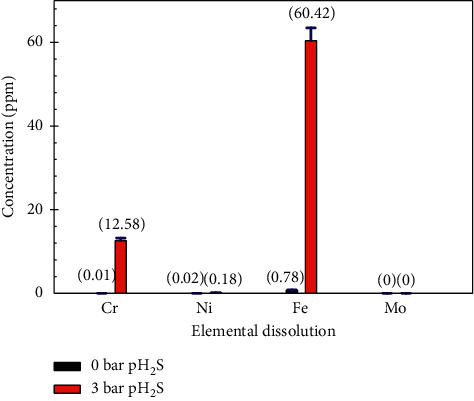
ICP-MS result of 316L austenitic stainless steels at two different conditions 0 bar and 3 bar partial pressure H_2_S in NACE A solution.

Metal ions of 0 bar and 3 bar pH_2_S after immersion in the test solutions for 7 days were evaluated via ICP-MS, as shown in Figure 7. *Y*-axis in Figure 7 shows metal ions concentration after 7 days immersion in the test solution, while *x*-axis shows the main elemental composition in the 316L steel with two plots for each element which represents a partial pressure of H_2_S. In brief, both solutions (0 and 3 bar pH_2_S) showed no Mo, but Fe and Cr were selectively dissolved. The highest concentration of Fe dissolved in the test solution suggests that Fe is the main component of the 316L stainless steel. The selective dissolution of Cr became more pronounced as 3 bar pH_2_S was exposed. One point to note is that metals that rely on Cr_2_O_3_ for corrosion protection may be at risk of having the Cr_2_O_3_ dissolve into the solution with increased pH_2_S. This issue is a considerable concern since an increase in the concentration of HS^−^, S^2-^, or Cl^−^ anions could accelerate the Cr_2_O_3_ film dissolution. In comparison to the 0 bar pH_2_S sample, the sample exposed to 3 bar pH_2_S had significantly increased the concentrations of Fe and Cr. Castle and Qiu (1990) [20] reported that Cr dissolves into solution as trivalent ions, while Fe dissolves as divalent ions. Additionally, the adsorbance of S on the metal surface produces metal sulfides that make the oxide film more defective [21]. Therefore, the values obtained from the ICP-MS results are generally in very good agreement with the XPS results when comparing the intensity of the composition of the compound on the sample surface to the dissolved metal ions in the test solution. The result showed that the depleted compound present in the corrosion product layer dissolved directly into the electrolyte during the experiment. Furthermore, the surface atomic percentage in Table 3 shows that the Cr content was very low for the sample exposed to 3 bar pH_2_S, indicating that Cr dissolution had indeed happened.

**Figure 8:**
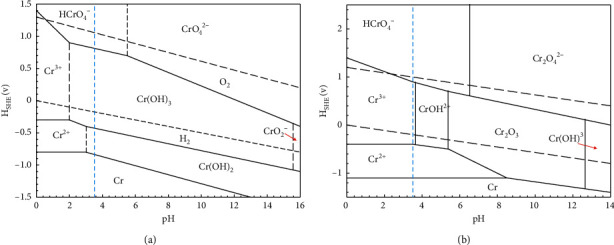
Pourbaix diagrams of Cr with (a) pH_2_S-free and (b) pH_2_S-containing conditions in aqueous at *T* = 25°C [22, 23].

Pourbaix [11] stated that Cr(II) ions are unstable and react very rapidly with oxygen in acidic solutions. Referring to the Pourbaix diagram, the formation of Cr, Cr(OH)_2_, Cr(OH)_3_, and HCrO_4_^−^ is related to the dissolution of Cr in H_2_O-Cl^−^ and H_2_O-S-Cl^−^ solutions, mostly in conditions close to pH 3.5 (blue dash line) with a potential range expected between 0 and 1 volt, as shown in Figures 8(a) and 8(b) [22, 23]. Overall, the dissolution process, which is the release of alloying elements in the electrolyte from the bulk or the film, was investigated using ICP-MS after 7 days of exposing the sample in NACE A solution. The main elements to dissolve were iron and chromium, which were detected in the electrolyte in the same proportion as the chemical composition of the 316L steel (Table 1).

### 3.5. Transmission Electron Microscopy (TEM)

**Figure 9:**
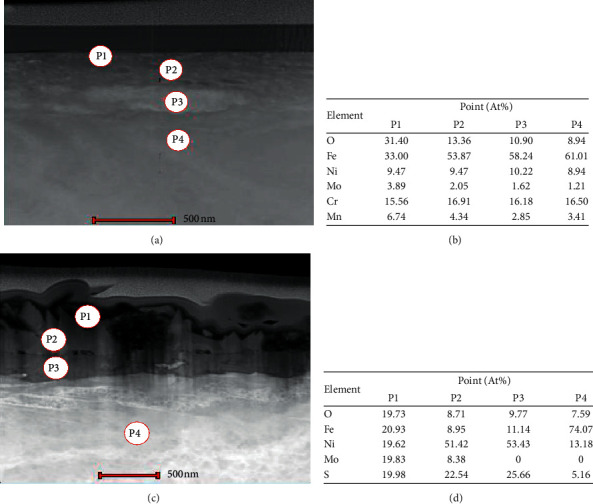
TEM images of corrosion layer on 316L austenitic stainless steel of 7 days immersion in NACE A solution: (a) 316L SS at 0 bar partial pressure H_2_S, (b) point EDS results for 0 bar partial pressure H_2_S, (c) 316L SS at 3 bar partial pressure H_2_S, and (d) point EDS results for 3 bar partial pressure H_2_S.

Transmission electron microscopy (TEM) was conducted to further investigate the structure of passive films. TEM micrograph with the point EDS of both 316L steels was recorded as shown in Figure 9. 316L steels were prepared using FIB with a platinum coating layer, an oxide layer, and a substrate of 316L steel. The layer formed in both samples showed different patterns and multilayered in the 3 bar pH_2_S 316L steel (Figure 9(c)) compared to that in the 0 bar pH_2_S 316L steel (Figure 9(a)). These films acted as layers that barred the reaction products from the corrosive environment and took the form of mono- or multilayer oxygen or other chemical species adsorbed onto the metal surface. The four-point EDS (marked as P1, P2, P3, and P4) was analyzed in both 0 bar and 3 bar pH_2_S conditions. P1 represents the outer layer/interface, P2 and P3 represent the inner layer, and P4 is the sample substrate. This analysis focused on elements that had a major contribution to the samples such as Fe, Ni, S, O, and Mo. Point 1 in the 0 bar pH_2_S sample comprised O, Cr, Mn, Fe, Ni, and Mo elements at the outer layer of the sample. However, Mn and Cr elements were not detected at P1 in 3 bar pH_2_S, but the S element was present in the outer layer of the 3 bar pH_2_S sample as shown in Figure 9(d). The Cr signal disappeared at the outer layer, due to formation of Cr(OH)_3_, hence suggesting that more Cr(OH)_3_ was dissolved in the test solution [24]. Thus, one of the primary elements that may contribute to P1 in the 3 bar pH_2_S is MoO_3_ and a result that is in good agreement with the XPS results. Moreover, points P2 and P3 represent the inner part of the multilayer that gives strong signals of Fe, Cr, and Ni for 0 bar pH_2_S and Fe, Ni, and S for the EDS analysis of 3 bar pH_2_S. In addition, Ni enriched about 51.42% at P2 and 53.43% at P3 for the 3 bar pH_2_S sample (Figure 9(d)). As the EDS and XPS results of 0 bar pH_2_S are linked, the primary elements that contributed to P2 and P3 were MoO_3_, MoO_2_, Cr_2_O_3_, and CrO_3_. However, it has been proposed that Mo could thicken the passive film, increase the surface affinity towards oxygen, and decrease the propensity for Cl^−^ to adsorb or form an extra secondary protective film [18]. Nevertheless, the primary elements that contributed to P2 and P3 at 3 bar pH_2_S were NiO, Ni(OH)_2_, NiS, FeS, Fe_3_O_4_, and FeS_2_, consistent with the EDS and XPS results. Since Ni did not participate in the passive film formation, it would be segregated by the oxides, and it consequently enriched underneath the passive film in the form of NiO and Ni(OH)_2_, as also verified by the XPS results in Figure 5(a). Finally, EDS P4 showed a strong signal attributed to the Fe element in both 0 bar and 3 bar pH_2_S conditions. The atomic composition showed Fe enriched by about 61.01% and 74.07% at 0 bar pH_2_S and 3 bar pH_2_S, respectively, both of which correspond to the bare metal.

**Figure 10:**
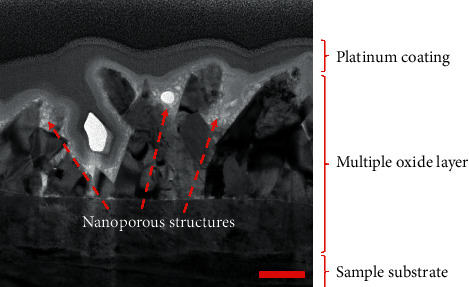
TEM image of 316L austenitic stainless steel in 3 bar partial pressure H_2_S, *T*_gas_ = 60˚C with nanoporous structure. Scale bar: 200 nm.

Meanwhile, the enlargement of TEM micrograph of 3 bar pH_2_S 316L steel in Figure 9(c) shows that the porousness of the oxide layer was precipitated at the upper layer of the film. Generally, passive films are formed with a highly disordered “barrier” layer adjacent to the substrate and film, comprising a precipitated phase that may incorporate anion and/or cation from the solution [25]. However, the contribution of ions in the NACE A solution, i.e., NaCl, CH_3_COOH, and H_2_O in the presence of H_2_S, affected the oxide layer of the 316L steel samples, forming multilayer corrosion products after 3 bar partial pressure H_2_S exposure as shown in Figure 10.

### 3.6. The Formation and Dissolution of 316L Corrosion Products

A passive film is envisaged to be a bilayer or multilayer structure comprising a point defective, nanocrystalline barrier layer and a porous outer layer that is formed by the hydrolysis of cations, depending upon the local conditions [25]. Corrosion products formation and dissolution are a continuous process and, generally, the film will have defects (vacancies and interstitials). At 3 bar pH_2_S condition, a decrease in corrosion resistance due to the introduction of sulfide ions (H_2_S, HS^−^, and S^2-^) to 316L steel was associated with the increase in proton reduction, leading to the anodic dissolution. Sulfides in the passive film are reported to have more defects than oxides [16], and thus, the film is less protective and stable in a high sour environment. In brief, Cr and Mo oxides were first generated under H_2_S-free conditions following the reactions in equations (2)-(3) for Mo oxides and the reactions in equations (4)–(6) for the Cr oxides:

(2) $$\begin{array}{c} {\text{Mo}}^{+}+{4\text{OH}}^{-}⟶{\text{MoO}}_{2}+{2\text{H}}_{2}\text{O} \end{array}$$

(3) $$\begin{array}{c} {\text{Mo}}^{6+}+{6\text{OH}}^{-}⟶{\text{MoO}}_{3}+{3\text{H}}_{2}\text{O} \end{array}$$

(4) $$\begin{array}{c} 2{\text{Cr}}^{3+}+6\text{OH}⟶2\text{Cr}{\left(\text{OH}\right)}_{3} \end{array}$$

(5) $$\begin{array}{c} 2\text{Cr}{\left(\text{OH}\right)}_{3}⟶{\text{Cr}}_{2}{\text{O}}_{3}+3{\text{H}}_{2}\text{O} \end{array}$$

(6) $$\begin{array}{c} {\text{Cr}}^{6+}+6{\text{OH}}^{-}⟶{\text{Cr}}_{2}{\text{O}}_{3}+3{\text{H}}_{2}\text{O} \end{array}$$

After long-time passivation, a stable passive film forms on the 316L steel surface with a dual-layer structure, as shown in Figure 9(a). The amorphous outer layer consists of CrO_3_, Cr_2_O_3_, MoO_3_, and MoO_2_ in H_2_S-free conditions, as proven by the XPS surface analysis (Figure 4). In addition, MoO_2_ and MoO_3_ metals are also formed after exposure to 3 bar pH_2_S via a reaction with hydroxyl, similar to the reactions in equations (2)-(3), respectively. The passivation of Mo at higher pH_2_S conditions could mainly be associated with the formation of MoS_2_ (equation (7)) [26]. However, the replacement of MoS_2_ by MoO_2_ could be predicted by the reaction with water as shown in equation (8) [26, 27]:

(7) $$\begin{array}{c} {\text{Mo}}^{4+}+4{\text{HS}}^{-}⟶{\text{MoS}}_{2}+2{\text{H}}_{2}\text{S} \end{array}$$

(8) $$\begin{array}{c} {\text{MoS}}_{2}+{2\text{H}}_{2}\text{O}⟶{\text{MoO}}_{2}+2{\text{H}}_{2}\text{S} \end{array}$$

Since the corrosion product of Cr compound did not form after 3 bar pH_2_S exposure due to dissolution of Cr in the test solution to produce Cr^3+^ (Figure 8), as per the reactions are shown in equations (9)–(11):

(9) $$\begin{array}{c} 2{\text{CrO}}_{3}+2{\text{OH}}^{-}⟶{\text{Cr}}_{2}{\text{O}}_{7}^{2-}+{\text{H}}_{2}\text{O} \end{array}$$

(10) $$\begin{array}{c} {\text{Cr}}_{2}{\text{O}}_{7}^{2-}+{\text{H}}_{2}{\text{O}+3\text{H}}_{2}\text{S}⟶2{\text{Cr}}^{3+}+{\text{H}}_{2}\text{O} \end{array}$$

(11) $$\begin{array}{c} {\text{Cr}}_{2}{\text{O}}_{3}+{\text{H}}_{2}{\text{O}+3\text{H}}_{2}\text{S}⟶2{\text{Cr}}^{3+}+{3\text{S}}^{2-}{+4\text{H}}_{2}\text{O} \end{array}$$

The depletion of Cr in the passive film resulted in the creation of a porous-precipitated layer because of the vacancies of cations and anions, as shown in Figure 10. Thus, depletion of Cr induces a higher S content in the passive film and results in passive film degradation. Besides, Ni(OH)_2_, NiO, and NiS are formed after exposure to 3 bar pH_2_S, respectively, based on the reactions in equation (12)–(14):

(12) $$\begin{array}{c} {\text{Ni}}^{2+}+2{\text{OH}}^{-}⟶\text{Ni}{\left(\text{OH}\right)}^{2} \end{array}$$

(13) $$\begin{array}{c} \text{Ni}{\left(\text{OH}\right)}^{2}⟶\text{NiO}+{\text{H}}_{2}\text{O} \end{array}$$

(14) $$\begin{array}{c} {\text{Ni}}^{2+}+2{\text{HS}}^{-}⟶\text{NiS}+{\text{H}}_{2}\text{S} \end{array}$$

Aside from that, Fe_3_O_4_, FeS, and FeS_2_ formations were slightly different compared to other elements in 3 bar pH_2_S conditions. The OH^−^ from the solution reacted first with ionic Fe in the outer passive film layer to form iron hydroxide followed by oxidation to form Fe_3_O_4_. The main reactions are described by the following equations [28]:

(15) $$\begin{array}{c} {\text{Fe}}^{2+}+{2\text{OH}}^{-}⟶\text{Fe}{\left(\text{OH}\right)}^{2} \end{array}$$

(16) $$\begin{array}{c} 4\text{Fe}{\left(\text{OH}\right)}^{2}+2{\text{H}}_{2}\text{O}+{\text{O}}_{2}⟶4\text{Fe}{\left(\text{OH}\right)}_{3} \end{array}$$

(17) $$\begin{array}{c} 2\text{Fe}{\left(\text{OH}\right)}^{3}+\text{Fe}{\left(\text{OH}\right)}_{2}⟶{\text{Fe}}_{3}{\text{O}}_{4}+4{\text{H}}_{2}\text{O} \end{array}$$

In an H_2_S environment, HS^−^ or S^2-^ could also incorporate into the film to form FeS and FeS_2_ based on the reactions in equations (18) and (19) [29]:

(18) $$\begin{array}{c} {\text{Fe}}^{2+}+{\text{S}}^{2-}⟶\text{FeS} \end{array}$$

(19) $$\begin{array}{c} {\text{Fe}}^{2+}+2{\text{HS}}^{-}⟶{\text{FeS}}_{2}+{\text{H}}_{2}{\text{Fe}}^{2+}+{\text{S}}^{2-} \end{array}$$

Therefore, the protectiveness of the 316L passive film could decrease, and the corrosion resistance of the 316L also deteriorates at higher H_2_S conditions. Hence, different materials could be subjected to further study and are analyzed at a range of different scales for safety concern, relating to the oil and gas production system.

## 4. Conclusions

From this study, the effect of H_2_S pressure on the formation of corrosion products on the 316L steels was investigated. The corrosion rate was increased at 3 bar pH_2_S, and the morphology of corrosion product experienced cracked surface and local breakdown of barrier layer compared to H_2_S-free conditions. Similarly, the TEM results have shown that the 316L steel at 3 bar pH_2_S condition exhibits more porous in the corrosion product layer. XPS results indicated the signals (peaks) of passive films which are Mo, MoO_3_, NiO, Ni(OH)_2_, NiS, Fe_3_O_4_, and FeS_2_. However, in H_2_S-free conditions, the only passive film was observed at MoO_2_, MoO_3_, Cr_2_O_3_, and CrO_3_ signals. Finally, Cr signals in XPS analysis were decreased after exposure to 3 bar pH_2_S, and it has been confirmed by ICP-MS that the chromium was dissolved into solution. Therefore, 316L could not be sustained in high partial pressure of H_2_S due to local breakdown of the crystalline layer, and the protectiveness of the passive film had very much degraded.

## Figures and Tables

**Table 1:** The chemical composition of the tested 316L austenitic stainless steel.

Composition	C	Si	Mn	P	S	Cr	Mo	Ni	Cu	Al	Fe
Weight (%)	0.02	0.43	1.31	0.03	0.01	16.48	2.08	10.18	0.34	0.01	Bal.

**Table 2:** The experimental parameters and weight loss of 316L steel exposed to two different environments.

Test condition	Without H_2_S	With H_2_S
Temperature (°C)	60	
Partial pressure H_2_S (bar)	0	3
Total pressure (bar)	30	

pH (before/after)	2.52/2.63	2.95/3.42
Weight loss (g)	0.01	0.086
Immersion time	7 days (128 h)	
Solution	NACE TM0177 solution A (5wt% NaCl and 0.5wt% CH_3_COOH in distilled water)	

**Table 3:** The XPS deconvolution of percentage surface atomic concentration for 316L steels at different conditions.

Condition	Surface atomic concentration %						
	O 1s	Cr 2p	Mo 3d	Fe 2p	Ni 2p	S 2p	C 1s
0 bar pH_2_S	26.31	1.74	0.20	—	—	—	Bal.
3 bar pH_2_S	34.52	0.10	0.19	0.20	6.94	1.15	Bal.

**Table 4:** The list of corrosion products from XPS deconvolution of percentage surface atomic concentration for 316L steels in different conditions.

Corrosion product	Binding energy (eV)	
	0 bar pH_2_S	3 bar pH_2_S
CrO_3_	578.48	Nil
Cr_2_O_3_	577.03	Nil
Mo	Nil	232.4
MoO_2_	228.85	Nil
MoO_3_	232.96	226.9
NiO	Nil	855.7
Ni(OH)_2_	Nil	857.4
NiS	Nil	853.9
FeS	Nil	712.8
Fe_3_O_4_	Nil	710.1

## Data Availability

The Pourbaix diagrams of Cr with pH_2_S-free and pH_2_S-containing conditions in aqueous at *T* = 25°C [23, 24] and data supporting (inductively coupled plasma-mass spectrometry (ICP-MS) analysis) are from previously reported studies and datasets, which have been cited. The processed data are available from the corresponding author upon request.
